# Crouzonodermoskeletal Syndrome with Hypoplasia of Corpus Callosum and Inferior Vermis

**DOI:** 10.4274/jcrpe.3343

**Published:** 2016-09-01

**Authors:** Fatih Gürbüz, Serdar Ceylaner, Ali Kemal Topaloğlu, Bilgin Yüksel

**Affiliations:** 1 Ankara Pediatric Hematology and Oncology Training and Research Hospital, Clinic of Pediatric Endocrinology, Ankara, Turkey; 2 Intergen Genetics Center, Clinic of Medical Genetics, Ankara, Turkey; 3 Çukurova University Faculty of Medicine, Department of Pediatric Endocrinology, Adana, Turkey

**Keywords:** Crouzonodermoskeletal syndrome, craniosynostosis, acanthosis nigricans, hypoplasia of corpus callosum, inferior vermis

## Dear Editor,

Crouzonodermoskeletal syndrome) [Online Mendelian Inheritance in Man (OMIM) ID no. 612247] or Crouzon syndrome with acanthosis nigricans (CSAN) is a clinically and genetically distinct entity from the classic Crouzon syndrome (1). While classic Crouzon syndrome is caused by mutation in the FGFR3 gene, CSAN is caused by only mutation p.A391E in the FGFR3 gene. Other FGFR3 gene mutations are not responsible of this syndrome, and they usually lead to hypochondroplasia or achondroplasia but not CSAN (2,3). Clinically, in addition to classic Crouzon syndrome, patients with CSAN have acanthosis nigricans and skeletal abnormalities (1,4). The phenotypic features include wide-set bulging eyes and underdeveloped upper jaw, craniosynostosis, midface hypoplasia, hypertelorism, proptosis, posteriorly rotated ears, and in some cases, hearing loss. Patients with CSAN often present with choanal atresia and hydrocephalus (4).

A ten-month-old girl presented with a facial dysmorphia at birth. There was no consanguinity between her parents. She was born at term weighing 3380 g with no perinatal complications. She had obstructive dyspnea at day 1 due to bilateral choanal atresia for which she was operated at day 9. At 9 months of age, she had coronal craniectomy surgery because of craniosynostosis due to bilateral coronal stenosis.

**Figure 1:**
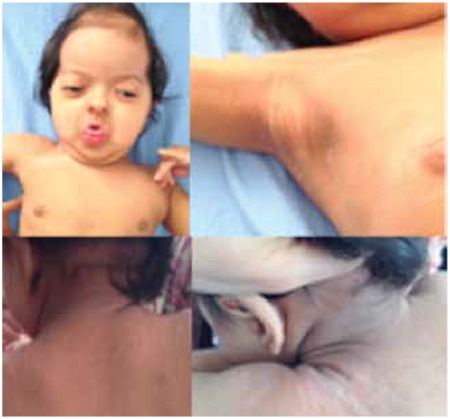
Patient’s facial dysmorphia and widespread acanthosis nigricans on neck and axillae

**Figure 2:**
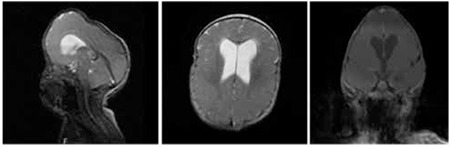
Patient’s magnetic resonance imaging: presence of hydrocephalus and hypoplasia of corpus callosum and inferior vermis

At presentation, her height was 68.7 cm [-1.13 standard deviation score (SDS)] and weight was 7.3 kg (-1.97 SDS). She had atypical facial features (midface hypoplasia, hypertelorism, craniosynostosis, brachycephaly, maxillary hypoplasia, exophthalmos, bilateral distinctive and low-set ears), lateral nystagmus on the bilateral eyes, and widespread acanthosis nigricans on all of curve regions as neck, bilateral axillae (Figure 1). Cranial magnetic resonance imaging (MRI) revealed hydrocephalus, hypoplasia of corpus callosum and inferior vermis (Figure 2). There was no pathology at abdominal ultrasonography and echocardiography. The audiogram did not reveal any pathology. The cognitive and motor development were delayed.

**Figure 3:**
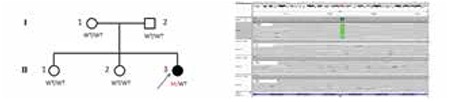
Sight of the patient’s pedigree and sequence (c.1172C>A)

We detected a de novo heterozygous A391E (c.1172C>A) mutation in FGFR3 gene in our patient. This syndrome is inherited in an autosomal dominant type although most cases are sporadic mutations (5). We detected the mutation in the patient but not in her parents and sisters (Figure 3). Therefore, our patient is a sporadic form of CSAN.

As with other disorders caused by FGFR gene mutations, increased paternal age seems to be a risk factor (1). Our patient’s father age was 42 years old.

To our knowledge, this is the first case of CSAN with hypoplasia of corpus callosum and inferior vermis. This association may be coincidental. These patients should be investigated for other possible cranial MRI findings.

## Ethics

Peer-review: Internal peer-reviewed.
